# Perceiving One’s Own Limb Movements with Conflicting Sensory Feedback: The Role of Mode of Movement Control and Age

**DOI:** 10.3389/fpsyg.2012.00289

**Published:** 2012-08-09

**Authors:** Lei Wang, Christine Sutter, Jochen Müsseler, Ronald Josef Zvonimir Dangel, Catherine Disselhorst-Klug

**Affiliations:** ^1^Department of Work and Cognitive Psychology, Rheinisch-Westfälische Technische Hochschule Aachen UniversityAachen, Germany; ^2^Department of Rehabilitation and Prevention Engineering, Helmholtz Institute of Applied Medical Engineering, Rheinisch-Westfälische Technische Hochschule Aachen UniversityAachen, Germany

**Keywords:** aging, visuomotor transformation, tool use, perception, action control, active and passive movement control, proprioception, vision

## Abstract

Previous studies have demonstrated a great uncertainty in evaluating one’s own voluntary actions when visual feedback is suspended. We now compare these limitations in younger and older adults during active or passive limb movements. Participants put their dominant hand on a robot arm and performed movements actively or the relaxed limb was moved passively. Either a distorted visual feedback or no visual feedback at all was provided during the movement. Perception of limb movements was attenuated through visual feedback. This effect was more pronounced in older adults. However, no difference between active and passive movements was found. The results provide evidence for the limited awareness of body effects, even in the absence of voluntary actions.

## Introduction

**Figure 1:**
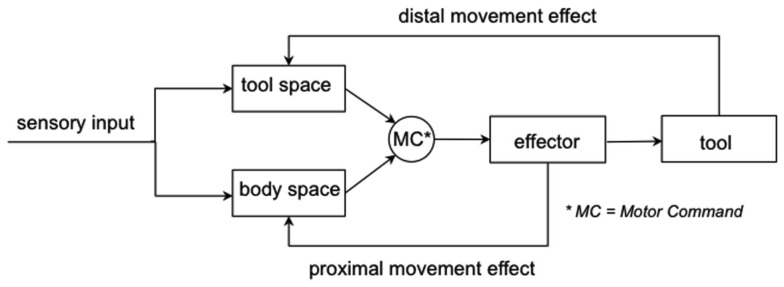
**The dual action-feedback loop of goal-oriented actions when using a tool**. The motor commands launch at first a movement of the body effector, which then causes the desired displacement of the tool. Thereby the actor receives the movement feedback from the own body (proximal movement effect) and from the tool (distal movement effect).

Intentional actions commonly generate bimodal sensory effects: on the one hand the proximal, body-related action effects like the proprioceptive sensation from the required joints, and on the other hand the distal action effects, for example, the displacement of the cursor on the monitor. These sensory inputs must be monitored and integrated for online action control and error-based learning, especially in case of tool use, as demonstrated in a dual-feedback model (Figure 1). The execution of motor commands produces spatial displacements of the body effector (e.g., the hand) and the tool (e.g., the mouse cursor on the computer screen) controlled by the body effector. Sensory feedbacks of proximal and distal movement effects will be used to update the actual spatial configuration of the body effector and the tool. Based on these updates, new motor commands will be generated to continue the action in a modified way.

The bimodal sensory inputs are not necessarily congruent. For instance, the hand movement controlling a computer mouse causes usually larger displacements of the cursor on the monitor. Since in most circumstances the distal goals of intentional actions are represented visually, visual information should be predominant. Direct evidence of visual predominance was first provided by Hay et al. (1965). In their study a wedge prism perturbed actual hand positions. As a result perceived hand positions shifted toward the visually displayed hand positions. This effect is one example of visual capture and verified through later investigation (e.g., Pavani et al., 2000). Further evidence was found in studies focusing on adaptive movement control (e.g., Bedford, 1993). The implementation of a visual distortion is one example for establishing a novel action environment in motor control. Exposure to such distortions, for example by introducing prism goggles (e.g., Bedford, 1993; Redding and Wallace, 1997, 2006), changes in visuomotor gain (e.g., Heuer and Hegele, 2007), or visuomotor rotation (e.g., Krakauer et al., 2000), lead to visuomotor adaptation. This reflects the flexible nature of the motor control system. Perceptual processes underlying such flexibility rely on the compliance of proprioceptive sensation. For instance, spatial re-alignment in prism adaptation is based on transformation of the proprioceptive mapping to match the changed visual mapping (Bedford, 1993; Redding and Wallace, 2006). Similar perceptual processes were observed by Ghahramani et al. (1996). In a pointing task participants adapted to perturbed visual feedback of the finger, so that actual finger positions arising from the proprioceptive sensation were remapped to the visually perturbed positions. Consequently, visual dominance and compliance of proprioception are fundamental for adaptive movement control in such cases. Furthermore, proprioception is even dispensable for adaptive control (Bernier et al., 2006) as demonstrated by a deafferented patient, who adapted to a novel kinematic environment in the same way healthy subjects did. Apparently, distal representations of the movement’s goal controls actions. In order to maintain the flexibility of the human information processing system (visual) distal action effects are predominant while proximal action effects are attenuated.

In addition, the proprioceptive sensation *per se* may be not as precise as the visual perception. In the experiment of Van Beers et al. (1998), participants were seated at a table and had to perform position-matching tasks relying either on visual or proprioceptive information. The precision of the visual localization was between 0.2° and 0.6°, whereas the proprioceptive position sense showed a larger variance ranging from 0.6° to 1.1°. Other studies also demonstrated a great uncertainty in perceiving one’s own voluntary actions when visual feedback was perturbed (Fourneret and Jeannerod, 1998; Slachevsky et al., 2001; Knoblich and Kircher, 2004; Müsseler and Sutter, 2009) or prohibited (Ghilardi et al., 1995). Taken together, the proprioceptive sensation of limb movements seems to be highly susceptible and less reliable than the visual sensation. Empirical evidence shows that humans are able to integrate multisensory signals in an optimized fashion to maximize the reliability of the perception (Ernst and Banks, 2002; Drewing and Ernst, 2006). Considering a motor action as an object of perception, integration of sensory feedback from visual and proprioceptive senses should follow the same principle. Therefore, in connection with the aforementioned lack of reliability of proprioception, it makes perfect sense that vision dominates action control, since the variance of the visual estimation is lower than that of the proprioceptive estimation.

The major question addressed in the current study is if there are any factors that moderate the bimodal integration, and consequently, affect the predominance of the visual feedback. We focused on two potential factors. A process-related factor could be the presence of motor commands. These can be understood as neural signals generated as exclusive sources of voluntary actions. Since motor commands build a link between distal and proximal action effects the movement mode should play an important role in information processing. The study by Zwickel et al. (2010) investigated whether producing active movements in a specified direction with a hand-held stylus or passive movements with the hand being transported by a robot affected the direction estimation of a concurrently presented stimulus motion. Judgments were significantly biased in the direction of the produced movement when movements were performed actively, whereas no such effect was observed for passive movements. Accordingly, we assume that the motor commands could enhance sensory integration and consequently strengthen the impact of distal feedback on proximal movement perception.

A subject-related factor could be age. Mounting evidence suggests that declines in proprioceptive function represent a fundamental aspect of the aging process (Adamo et al., 2007; Ribeiro and Oliveira, 2007; Goble et al., 2009). A variety of age-related neurophysiological changes may account for the declines in proprioception. Changes in the peripheral nervous system as potential cause are for example decreased spindle diameter, decreased sensitivity of muscle spindles, decreased number of intrafusal fibers, and a decline in the number of joint mechanoreceptors (for a comprehensive review see Goble et al., 2009). Declines in proprioceptive functions are also thought to be a result of changes in the central nervous systems, since increased proprioceptive processing demands were found to significantly impact the assessment of proprioceptive acuity in the elderly (Stelmach et al., 1990; Teasdale and Simoneau, 2001; Adamo et al., 2007). Based on these findings, we assume that the elderly would be more dependent on the visual feedback, which would then unfold its dominance more intensively.

Finally, the following hypotheses were proposed: (a) Distorted visual feedback makes movement perception more difficult. (b) Compared with younger people the older participants should show a poorer performance in perceiving their own body movements. (c) The impact of distorted visual feedback should unfold more intensively for older people. (d) Active movements should enhance the impact of the distorted visual feedback and cause poorer performance in both age groups. To examine these hypotheses, the current study compared the performance in limb movement perception of older and younger adults in various feedback (distorted visual feedback vs. no visual feedback) and movement control (active vs. passive movement execution) conditions.

## Materials and Methods

### Participants

In total eight younger participants (five male), aged between 22 and 29 years (mean: 25 years; SD: 2.7 years) and eight older participants (four male), aged between 61 and 70 years (mean: 66.5 years; SD: 4 years) voluntarily participated. The younger participants were students of the RWTH Aachen University. The older participants were recruited from the senior-college of the RWTH Aachen University via phone calls. All of them were right-handed and had normal or corrected-to-normal vision. Participants were all neurologically intact and had no known history of neuromotor disorders. Prior to the experiments, participants signed an informed consent statement.

### Apparatus and stimuli

**Figure 2:**
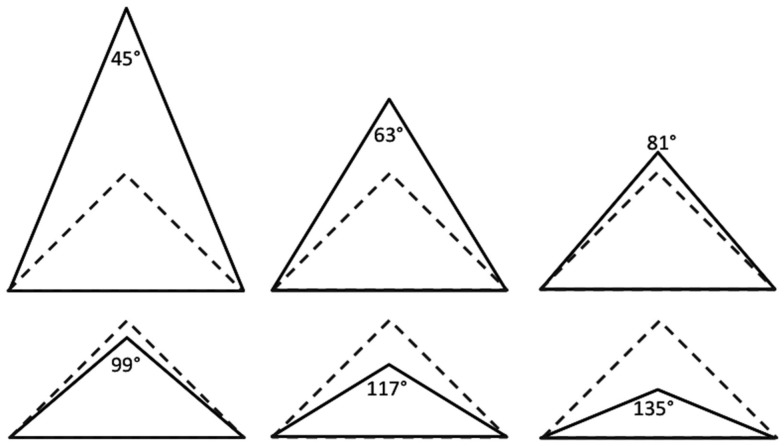
**The six standardized movement trajectories of the hand (solid lines)**. For visual feedback an equal-sided right-angled triangle (with dashed lines) was constantly displayed.

The experiment was carried out in a movement analysis laboratory using a lightweight robot LBR-IV. It belongs to a new generation of robots developed first by the German Aerospace Center (DLR). The robot presents redundant kinematics with seven degrees of freedom, allowing more complexity in the execution of the movements. Sensors evaluating the torque in each joint in real-time provide several useful features, for instance the compensation of the gravity and accelerated reaction when the robot is submitted to external forces. The robot LBR-IV was deployed to define six standardized trajectories (Figure 2, solid lines) that formed either an acute (*g* = 45°, 63°, or 81°) or an obtuse triangle (*g* = 99°, 117°, or 135°). All triangles were isosceles with a constant horizontal base of 26 cm.

**Figure 3:**
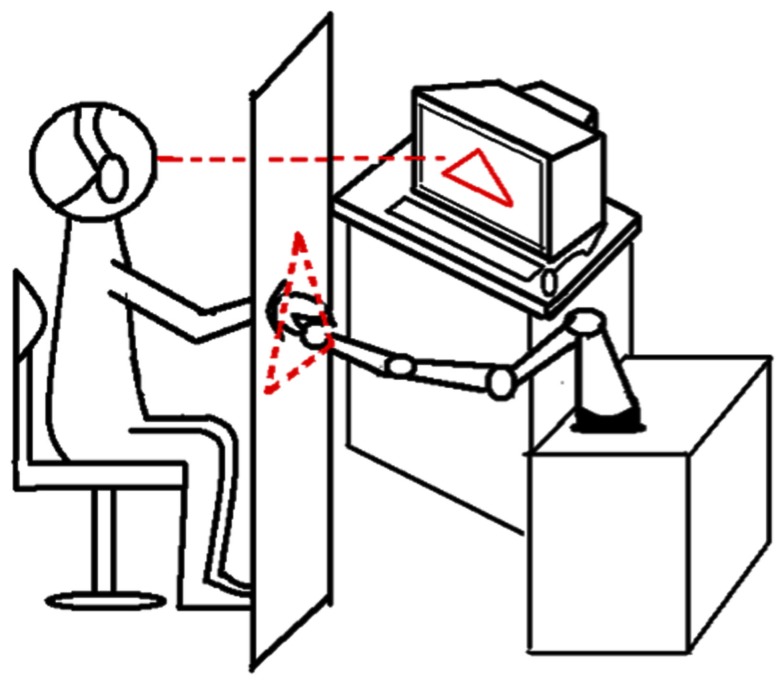
**Schematic view of the set-up**. The participant is sitting in front of a curtain, putting her/his hand through it on a robot arm. With help of the robot, limb movements could be carried out passively or actively. A distorted visual feedback about the limb movement could be presented on a LCD monitor, left alongside the robot in front of the participant.

The participants sat on a chair in front of the robot arm (Figure 3). The chair and the robot arm stood immovable through the experiment. The right shoulder of the participant and the resting robot arm on its start location were on the same sagittal plane. The distance between the shoulder of the participant and the robot arm was approximately 70 cm. Participants put their dominant hand on the robot arm and either performed the movement actively or the relaxed limb was moved passively. Every movement was constrained within one of the six pre-defined trajectories. Short audio signals (pure tone with 840 Hz for 100 ms) were provided to mark the beginning and the end of each movement. The audio signals were clearly audible to the participants, despite the ear protection they were wearing throughout the experiment. The actual limb movement was covered by a curtain (2 m × 1.6 m) and thus, invisible to the participants. During the movement the participants either received distorted visual feedback on a LCD monitor (Eizo FlexScan L768, 19″, 75 Hz refresh rate, 1024 × 768 pixel resolution), which was positioned approximately 110 cm away and 30° left in front of the participants, or no visual feedback at all. The distorted visual feedback consisted of a cursor (a blue dot with a diameter of 3 mm) moving along the sides of a static equilateral right-angled triangle with a base of 26 cm (Figure 2, dashed lines), which was presented centrally on the display.

Communication between the robot arm and the feedback monitor was facilitated by a MatLab (R2009a) program on a Windows computer. The movement of the robot arm was proportionally transferred into cursor movement, depending on the ratio between the total length of the actual limb trajectory and the feedback triangle, so that the cursor appeared to be completely synchronized with the robot arm. The passive movements have pre-defined acceleration profiles and a constant duration of approximately 6 s. In order to ensure that participants in the visual feedback condition were really tracking the cursor as instructed, 10% of the trials were constructed as so-called catch trials. In catch trials the blue cursor indicating the movement brightened shortly (yellow), which should be detected and reported by the participants as a secondary task. Immediately after the completion of the movement they had to give a verbal response according to their estimation of the shape of the hand trajectory by saying “spitz” (=“acute”) or “stumpf” (=“obtuse”). The experimenter registered the responses manually.

### Procedure

The experiment was carried out in two consecutive sessions. In Session I, a trial started with an audio signal, after which the robot arm began to move. Starting from the vertex down left, the robot led the relaxed right hand of the participant to complete one of the six standardized trajectories. After another audio signal indicated the end of the movement, participants instantaneously estimated the shape of their unseen hand trajectory.

Session I contained two blocks differing in feedback conditions: one block contained only trials with distorted visual feedback, while the other block contained only trials without visual feedback. The sequence of the blocks was counterbalanced across all participants. Every pre-defined trajectory was presented 15 times resulting in 90 trials per block. Prior to experimental trials 15 practice trials were provided to familiarize the participants with the task and its requirements. The whole session took about 60–70 min. At the end of the session participants were given a short questionnaire, in which they were asked about the strategy for making their estimations.

Session II followed the same procedure as Session I, except for the movement mode. Instead of being passively moved by the robot arm (Session I), participants had to accomplish the movement actively by pushing the robot along the pre-defined trajectories. Session II was carried out at least 6 weeks later than Session I. This quite long interval was introduced to avoid transfer effects from the preceding session.

### Data analysis

Hit rates were computed by coding the binary judgments as either correct or incorrect and calculating the percentage of correct answers. For hit rates (percentage) a 2 × 2 × 2 × 6 mixed ANOVA with the between-subject factors age (young vs. older) and movement mode (passive vs. active), and the within-subject factors feedback (distorted visual feedback vs. no visual feedback) and shape (45°, 63°, 81°, 99°, 117°, and 135°) was conducted. The second dependent variable was the area under the curve (AUC). Given the binary nature of the behavioral data and perceptual sensitivity as the underlying ability dimension, we computed a direct indicator for the perceptual sensitivity relying on receiver operating characteristic (ROC). This method is based on the signal-detection theory (for a review see Macmillan and Creelman, 2005). It provides a possibility to estimate the true sensibility of the participants, which is independent of their individual and often varying decision criteria. AUC reaches the maximum of 1, when judgments are perfect and without any error; AUC has the minimal value of 0, when the judgments are made completely randomly. Based on aggregated judgments across all stimuli, the mean AUC of ROC was calculated for each participant in each feedback and movement condition. For mean AUCs a 2 × 2 × 2 mixed ANOVA with the between-subject factors age (young vs. older) and movement mode (passive vs. active), and the within-subject factor feedback (distorted visual feedback vs. no visual feedback) was conducted.

## Results

**Figure 4:**
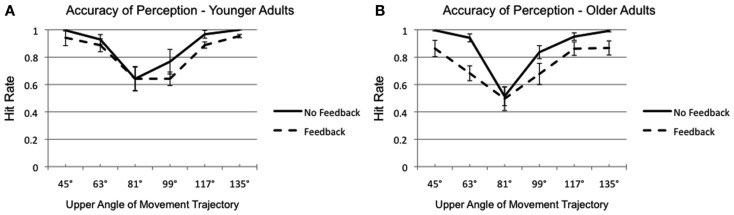
**Hit rate with (dashed line) and without (solid line) visual feedback of younger (A) and older (B) participants**. Each data point represents the average of all repetitions of a certain movement trajectory. The error bars represent the standard errors.

Results regarding the hit rates (percentage of correct answers) showed that the accuracy of participants systematically varied with the shape of the trajectories. The stronger a movement trajectory deviated from a right-angled triangle, the easier it was for the participants to judge the movement correctly (Figure 4). Overall performance across all stimuli indicated that participants were remarkably uncertain about their own hand movement, especially when the distorted visual feedback was presented. The average hit rate in this condition did not exceed 77% across all stimuli, and was 10% lower than the hit rate without visual feedback. In accordance with the aforementioned comparison between both feedback conditions, a significant main effect of the factor feedback was found [*F*_(1,14)_ = 19.68, *p* < 0.001, η^2^ = 0.58]. The trajectory shape (different triangles) also influenced the hit rate significantly [*F*_(5,70)_ = 21.17, *p* < 0.001, η^2^ = 0.60]. And more importantly, a trend of the feedback by age interaction was observed [*F*_(1,14)_ = 3.09, *p* < 0.10, η^2^ = 0.173], which was caused by a stronger decline in performance of older adults due to the distorted visual feedback. No other discernable effects were found in the ANOVA, which means that the expected main effects of age and movement mode were not observed.

**Figure 5:**
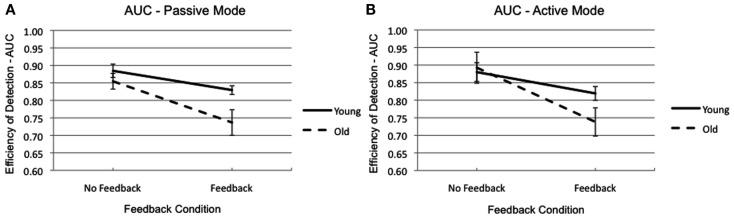
**The AUC of younger (solid line) and older (dashed line) participants depending on feedback condition and movement mode [(A) passive and (B) active]**. The error bars represent the standard errors.

Based on our hypotheses, the sensitivity of the participants, and therefore the AUCs should be influenced by feedback, movement mode, and age of the observer. The disturbance through visual feedback was statistically significant [*F*_(1,14)_ = 21.18, *p* < 0.001, η^2^ = 0.60], indicating a poorer sensibility with distorted feedback (*M* = 0.78) than with no feedback (*M* = 0.88). As depicted in Figure 5, the impact of distorted visual feedback was tendentially more manifest in older adults than in younger [feedback by age interaction: *F*_(1,14)_ = 3.45, *p* < 0.084, η^2^ = 0.20]. This finding is corroborated through independent sample *t*-tests (with Bonferroni correction, α_adjust_ = 0.025), yielding a tendency for a difference between younger and older participants [*t*_(14)_ = 1.83, *p* < 0.045, one tailed], when distorted visual feedback was given. All other main effects (including the expected main effects of age and movement mode) and interactions were not significant (*p* > 0.10).

## Discussion

The present study aimed to examine the predominance of visual perception over proprioceptive perception of limb movement in different conditions. Binary judgments regarding movement shape turned out to be less accurate when distorted visual feedback was presented during movement execution. This impact on performance was tendentially stronger in older participants than in the young ones. No difference was observed between active and passive movement execution. The issue of age-related changes and results regarding the factor movement execution will be discussed.

### Age-related changes in action control and perception

In our study, a significant influence of distorted visual feedback on movement perception was observed. The attenuation of proximal action effects was in accordance with our assumption. This influence was obtained for both age groups and had tendentially a greater impact on the older participants. This is in accordance with our assumption that in case of distorted visual feedback the older participants should rely more on visual information, which provides apparently more reliable information and causes stronger visual capture. However, the absence of group differences in the condition without visual feedback suggests that age-related degeneration in peripheral neural structures alone cannot account for the result. As mentioned earlier, attentional processes may have played a crucial role as well. Age-related deficits in position sense, motion sense, and dynamic position sense would increase the demand for proprioceptive movement monitoring (Seidler-Dobrin and Stelmach, 1998). Consequently, the interplay of increased demand to process proprioceptive information, the decreased attentional resources in elderly (e.g., Doumas et al., 2008), and attentional distraction through visual feedback may have resulted in tendentially poorer performance of the older participants. This finding could be a possible explanation for the lack of explicit strategic action control in elderly (McNay and Willingham, 1998; Hegele and Heuer, 2010), since knowledge about discrepancy between visual and proprioceptive information about the movement is indispensable to generate appropriate control strategies.

In the absence of visual feedback, the older participants showed nearly identical performance to the young ones. This finding was not in line with a multitude of previous studies investigating the relationship between aging and motor ability (Darling et al., 1989; e.g., Cooke et al., 1989; Boisgontier et al., 2012). These studies indicated a clear decline of proprioceptive acuity in the elderly. For example, Adamo et al. (2007) demonstrated that reproducing elbow joint positions relying only on the proprioceptive sense resulted in significantly poorer performance in older adults than in younger adults. There are several methodological reasons that could account for the absence of the expected effect related to age.

First, the task used in the current study differed substantially from those of previous studies (e.g., Stelmach and Sirica, 1986; Pickard et al., 2003; Adamo et al., 2007, 2009) examining the sense of limb position across the lifespan. These studies employed typically single joint matching tasks where the participants were required to match a memorized target joint angle in the absence of vision or to match a concurrently held limb position with the contralateral limb. In the current study the task required a binary judgment rather than a position match. The task required participants to monitor and to reconstruct the movement trajectory based on crucial movement segments, which concurrently recruited multiple joints (shoulder, elbow, and wrist). Reproducing a position may be a much more sensitive measure than giving a binary judgment. Additionally, the older participants in the current study were 66 years on average. Hence, they belong to adults at late working age. It has been argued that age-related changes in proprioception, especially in upper limb position sense are more pronounced in individuals exhibiting a sedentary lifestyle (Adamo et al., 2007), which is apparently not the case for our older participants who were students at the senior-college. Indeed, all older participants in our study reported in a pre-experimental survey that they frequently use a computer and can handle a computer mouse skillfully. It can be assumed that declines in proprioceptive functions may generally represent a fundamental aspect of the aging process, however, behavioral decline will not manifest strongly in adults at late working age, especially when they practice an active lifestyle. Taken together, the task used in the current study was probably not sensitive enough to detect age-related differences. Therefore, it remains interesting to replicate the study with participants of higher seniority and to measure additional behavioral indicators like movement reproduction.

Second, due to the small sample size the current study may have a lacked power. Evaluation of the short questionnaire, to inspect the individual judgment behavior, revealed a noticeable diversity of strategies. The participants seemed to have used very different movement cues to inform their judgments. These cues could be simple, e.g., “the height” and “the side length” of the triangle, or they could be more complex, e.g., “the ratio between height and base.” Some cues were even dynamic, for instance “the acceleration at the first ascent.” And some participants seemed to switch between strategies in different conditions. The large variety of strategies could have increased the variance in judgments. As depicted in Figure 4, the data regarding hit rates showed large variances across participants. This could have covered the age-related effects. Taking the factor age for example, the ANOVA reported in the early section yielded a *p* = 0.139 and an observed power (1 − β) of 311, which apparently had a substantial scope for improvement with a larger sample size. Due to technical restrictions an increase of test sample was not possible for the current study. Nevertheless, we believe that the preliminary data of the current study will be confirmed by a future work with an optimized sample size.

### Active vs. passive movement

The dual action-feedback loop (Figure 1) suggests the necessity for the motor system to integrate bimodal feedbacks in order to control voluntary actions. Consequently, the perception of one’s own limb movement is attenuated by the distal action-feedback. More importantly, the stronger the integration is, the larger the influence of visual feedback could be. Since Zwickel et al. (2010) showed that active movements could substantially enhance bimodal integration, we assumed that active movements should strengthen the impact of distorted visual feedback and cause poorer judgment performance compared to passive movements.

Contrary to our prediction, the mode of movement execution did not show any influence on the judgment. In this context, it is important to take a more comprehensive view on potential effects of active movements. On the one hand the efference copy of motor commands can directly contribute to the human-position sense (Winter et al., 2005; Gandevia et al., 2006; Gritsenko et al., 2007), and on the other hand active movement control can contribute to human-position sense by improving proprioception (Laufer et al., 2001). These findings would however lead to a contradictory prediction as we have originally made, namely improved judgment performance with active movement execution. Since the variation of movement mode did not cause any changes in the performance, it is not clear whether the mechanisms canceled each other, or rather there was no effect of the movement mode at all. The latter possibility could be due to the particular feature of the active movements in the current study. The active movement mode allowed the participants to move their dominant hand actively, however, these active movements differ from real goal-directed actions in at least two aspects. Firstly, the control of the own movement was limited to velocity and acceleration. Secondly, instead of one smooth aiming movement there were three single movement segments, one segment along each side of the triangle. Thus, constrained active movement represents only an intermediate level of motor control between pure passive movement without any control and pure voluntary action and therefore could be insufficient to enhance bimodal integration and the crosstalk of visual and proprioceptive sense. This speculation could be examined in a future study by comparing different movement modes regarding both visual capture of the proprioceptive position sense, and conversely, the repulsion effect of actual body movement on the visual perception (Zwickel et al., 2010).

## Conclusion

The purpose of the present study was to examine the predominant role of distal feedback in both active and passive movement modes and in younger and older adults. The results supported previous observations about the limited awareness of the proprioceptive sense, and more importantly, evidence for those limitations even in the absence of voluntary actions was provided through the present study as well. Although there was a slightly stronger interference from distal action-feedback for our older participants, it is worth stressing that they, despite of expected age-related functional declines, did not show any noticeable difference in their performance compared to the younger adults, at least if there was no distracting visual feedback.

Since the coordination of perception and action is a major function in human information processing and a pre-requisite for successful interactions with our environment, it is substantial to understand how humans integrate all the information from various senses to perceive their own actions and to act adequately. Further investigations based on our findings could provide an empirical basis for various applied fields, especially for the design of tools and working environments, in which sensorimotor transformations are essential.

## Conflict of Interest Statement

The authors declare that the research was conducted in the absence of any commercial or financial relationships that could be construed as a potential conflict of interest.
